# A Multiple Technology–Based Physical Activity Intervention for Latina Adolescents: Results From the Chicas Fuertes Randomized Controlled Trial

**DOI:** 10.2196/71623

**Published:** 2025-12-11

**Authors:** Jacob R Carson, Emily Greenstadt, Brittany Olivera, Shira Dunsiger, Michelle Zive, Michael Higgins, Job Godino, Bess Marcus, Dawn Meyer, Britta Larsen

**Affiliations:** 1 Herbert Wertheim School of Public Health and Human Longevity Science, University of California, San Diego, 9500 Gilman Dr, La Jolla, CA, 92037, United States, 1 9512952040; 2 Department of Behavioral and Social Sciences, Brown University, Providence, RI, United States; 3 Exercise and Physical Activity Resource Center, University of California, San Diego, La Jolla, CA, United States; 4 Laura Rodriguez Research Institute, Family Health Centers of San Diego, San Diego, CA, United States; 5 Department of Neurosciences, School of Medicine, University of California, San Diego, La Jolla, CA, United States

**Keywords:** mHealth, health disparities, Fitbit, Latina adolescents, physical activity, mobile health

## Abstract

**Background:**

Latina adolescents report low levels of moderate-vigorous physical activity (MVPA) and high lifetime risk of lifestyle-related diseases. There is a lack of MVPA interventions targeted at this demographic despite documented health disparities. Given their high rates of mobile technology use, interventions delivered through mobile devices may be effective for this population.

**Objective:**

This paper examines the efficacy of the Chicas Fuertes intervention in increasing MVPA across 6 months in Latina adolescents.

**Methods:**

Participants were Latina adolescents (aged 13‐18 years) in San Diego County who reported being underactive (<150 min/wk of MVPA). All participants received a wearable fitness tracker (Fitbit Inspire HR); half were randomly assigned to also receive the multimedia intervention. Intervention components included a personally tailored website, personalized texting based on Fitbit data, and social media. The primary outcome was change in minutes of weekly MVPA from baseline to 6 months, measured by ActiGraph accelerometers and the 7-Day Physical Activity Recall Interview. Changes in daily steps using Fitbit devices were also examined to test intervention efficacy.

**Results:**

Participants (N=160) were 15.85 (SD 1.71) years old on average, and mostly second generation in the United States. For ActiGraph-measured MVPA, participants in the intervention group (n=83) increased from a median of 0 (IQR 0-24) minutes/week at baseline to 64 (IQR 19-72) minutes/week at 6 months compared to control participants, who showed increases from a median of 0 (IQR 0-26) at baseline to 41 (IQR 7-76) minutes/week at 6 months (*P=*.04). Self-reported MVPA increased in the intervention group from a median of 119 (IQR 62.5-185) minutes/week at baseline to 147 (IQR 96-181) minutes/week at 6 months compared to control participants, who showed increases from a median of 120 (IQR 48.8-235) at baseline to 124 (IQR 100-169) minutes/week at 6 months (*P*=.03). Steps also increased in both groups, with the intervention group showing significantly greater increases (*P*=.03).

**Conclusions:**

This intervention was successful in using a tailored technology-based strategy to increase MVPA in Latina adolescents and provides a promising approach for addressing a key health behavior. Given the scalable technology used, future studies should focus on broad-scale dissemination to address health disparities.

## Introduction

Physical activity (PA) declines by as much as 90% between early childhood and adolescence, particularly in girls [1]. Additionally, adolescent girls of racial and ethnic minorities have the lowest PA rates of any demographic group in the United States [1-3]. According to the National Health and Nutrition Examination Survey, only 2.9% of Mexican American girls meet the national guidelines of 60 minutes of moderate-vigorous physical activity (MVPA) per day [1]. Increasing MVPA in Latina adolescents could improve physical, psychosocial, and cognitive health [4] while producing lifelong habits that mitigate obesity, type 2 diabetes, and other chronic illnesses that disproportionately impact Latina adults [5-7]. To date, no large-scale interventions focused on increasing MVPA in Latina adolescents are documented in the scientific literature.

Previous literature has identified that psychosocial variables may be responsible for PA disparities in Latina teens [8-16]. Adolescent girls frequently report lower self-efficacy, social support, and behavioral capability than boys [8,14-17], and Latina teens specifically report lower positive support and more negative support than non-Latina White girls [10,11]. An intervention grounded in theory to promote psychosocial constructs such as self-efficacy, social support, and perceived competence could be effective for increasing MVPA. Additionally, a previous meta-analysis found the strongest effects on PA for multicomponent interventions that were specifically targeted at adolescent girls (as opposed to adolescents generally) [18]. To date, employment of these intervention strategies in cohorts of Latina adolescents has been limited, specifically interventions that deliver via newer technologies [19].

We conducted a pilot study, Niñas Saludables*,* to test a web-delivered theory-based MVPA intervention for Latina teens, adapted from an intervention shown to be effective with Latina adults [20]. Self-reported MVPA increased significantly, and the intervention was generally well-received. In follow-up interviews, however, participants highlighted a desire for more visual intervention content in smaller, more frequent doses and the use of more mobile technology channels. This was corroborated in additional focus groups and design workshops, in which Latina teens highlighted a desire for an intervention designed specifically for them, along with use of wearable trackers and social media [21].

The aim of the Chicas Fuertes trial was to test the efficacy of a theory-based, individually tailored, multitechnology intervention for increasing MVPA in Latina adolescents compared to a control group receiving a wearable tracker. Descriptions of the intervention development, study protocol, recruitment strategies, and baseline findings have been published previously [22]. This paper presents the main findings for the Chicas Fuertes trial, which examined changes in MVPA from baseline to 6 months measured by the ActiGraph accelerometer and 7-Day Physical Activity Recall (PAR) Interview, and steps measured by the Fitbit Inspire HR monitor.

## Methods

### Overall Design

The Chicas Fuertes trial tested an individually tailored, multitechnology intervention aimed at promoting MVPA among Latina adolescents (N=160) in San Diego County. Promotion of MVPA over total activity or light physical activity (LPA) was prioritized to align with current PA guidelines [23]. The intervention is based on multiple behavior change theories, including social cognitive theory [24], the transtheoretical model [25,26], Control Theory, and the Behavior Change Techniques taxonomy [27]. Components of theory integrated into the intervention design included goal setting, self-monitoring, problem-solving barriers, increasing social support, social norms, and rewarding oneself for meeting goals. Intervention components were delivered via multiple media channels over 6 months. The primary outcome was change in minutes of weekly MVPA from baseline to 6 months, measured by ActiGraph accelerometers (objective) and the 7-Day PAR Interview (self-report). Psychosocial and environmental variables were also measured at baseline and follow-up.

### Study Population

To participate, individuals had to self-identify as Latina; be 13‐18 years old; live in San Diego County; read, write, and speak in English; and be underactive (ie, self-reporting less than 150 weekly minutes of MVPA). Additionally, participants needed regular (≥2 times/wk) access to the internet and access to a cell phone; participants who did not have a personal smartphone were provided additional equipment to sync their Fitbit to a computer and access Fitbit through a website instead of using the smartphone app. Participants were excluded if they self-reported a serious heart condition, chest pain during exercise, pregnancy, uncontrolled asthma, or any other serious medical condition that would make unsupervised PA unsafe, unless they provided written permission to participate from their health care provider. Strategies for recruitment, randomization, allocation, and blinding have been published previously [22,28].

### Measures

#### Primary Outcome: PA

##### ActiGraph Accelerometer

MVPA was measured objectively at baseline (prior to randomization) and 6-month follow-up using the ActiGraph GT3X+ accelerometer (ActiGraph, LLC). The ActiGraph GT3X+ is a hip-worn triaxial accelerometer worn on the hip that measures the movement and activity intensity. It has been validated against heart rate telemetry [29] and total energy expenditure [30], including in children [31]. Accelerometers were delivered via mail with detailed instructions to wear the accelerometer around their waist (as a belt) on their left-side hip for an entire day (approximately 12 hours) for 7 consecutive days. Throughout the week, participants received SMS reminders to wear the ActiGraph. Participants were provided with a prepaid and preaddressed envelope to mail back the accelerometer after use, at which time it was checked for sufficient wear time. Valid wear time was defined as wearing the device for at least 600 minutes per day for 5 days or a total of at least 3000 minutes over 4 days, and participants were instructed to rewear the device if they did not meet the criteria. Activity was considered part of the total minutes per week only if it occurred in bouts of 10 minutes or longer. The same procedure was followed for the 6-month follow-up visit.

##### 7-Day PAR Interview

The 7-Day PAR, a semistructured interview-administered instrument that assesses the frequency, duration, and intensity of MVPA, served as an additional primary outcome at baseline and 6-month follow-up [32]. The 7-Day PAR Interview asks participants about their activities over the past week and their estimates of their weekly PA in minutes. To improve recall accuracy, it breaks down activities by time of day, asks about the intensity and the purpose of the activity (eg, transportation, leisure, occupation, or school). It has been validated in children as young as age 11 years [33], and consistently shown acceptable validity and internal consistency [32,34], as well as sensitivity to changes over time [35,36]. The 7-Day PAR also measures sleeping time, work hours, and school hours to enhance accuracy. After wearing the accelerometer for a full 7 days (with valid wear time), participants were asked to complete the 7-Day PAR Interview to coincide with the accelerometer wear. All 6-month follow-up PAR interviews were conducted via Zoom (Zoom Communications) video.

##### Fitbit Inspire HR

Each participant enrolled was provided a Fitbit Inspire HR to be worn daily throughout the study period. Fitbit monitors were worn on the nondominant wrist and continuously captured day-level (steps) and minute-level (activity intensity and heart rate) data, in addition to sleep. The Fitbit app (Fitabase) automatically syncs with the monitor and stores historical data and offers a user-friendly interface to review activity and set goals for the participant. The Fitbit has excellent validity for measuring steps in free-living conditions [37]. Fitbit automatically classifies the intensity of activity based on movement and heart rate. Classification of MVPA has shown moderate validity, and recent studies have shown a high correlation between MVPA measured by the Fitbit and ActiGraph accelerometers [38]. To prevent feedback from influencing baseline activity, participants’ Fitbits were initially blinded with tape over the display and no activity information was accessible on the Fitbit app.

Each participant’s Fitbit accounts were linked to Fitabase (Small Steps Labs LLC), a third-party research platform that collects continuous Fitbit data whenever data are synced with the Fitbit app. An adherent day was characterized by having either 10 or more hours of heart rate data or 6000 or more steps [22,39]. Participant-level data for analysis were derived by averaging daily summaries across all adherent days.

### Secondary Outcomes and Covariates

Prior to their baseline and 6-month visits, participants received a personalized link to complete questionnaires directly into REDCap (Research Electronic Data Capture; Vanderbilt University) software, a Health Insurance Portability and Accountability Act (HIPAA)-compliant clinical trials software database. They were sent 3 reminder emails if the surveys were not completed by the time of the follow-up visit.

The 5-item Self-Efficacy for Physical Activity Scale was used to measure self-efficacy to become physically active across diverse contexts (α=0.82) [40]. We also administered the Social Support for Exercise Scale, which is a 13-item questionnaire with the 3 subscales of family, friends, and rewards and punishments focused on social support in the last 3 months (α=0.61‐0.91) [41]. To assess enjoyment for PA, the 18-item Physical Activity Enjoyment Scale (PACES) is used to evaluate the level of personal satisfaction from PA (α=0.96) [42]. Neighborhood environment is measured using the Neighborhood Environment Walkability Scale for Youth (NEWS-Y) and the Physical Activity Neighborhood Environment Scale (PANES). The NEWS-Y measures perceptions of neighborhood environment across 67 items across 9 subscales, including recreation facility availability, pedestrian traffic safety, and walking facilities having shown acceptable internal consistencies and correlations with objectively measured PA [43]. The PANES is a 9-item questionnaire including Likert responses to questions about walking, cycling, and safety features to indicate the neighborhood environment of participants [44]. Finally, participants also filled out the Center for Epidemiological Studies Depression Scale (CES-D) at baseline and the 6-month follow-up visit. The CES-D is a 10-item questionnaire related to depression symptoms experienced by the participant over the past week [45].

Two questionnaires were administered to everyone at baseline and monthly to the intervention group to develop tailored content for the website (see “Tailored Mobile Health PA Intervention (Intervention Group)”). Stages of Change for Physical Activity is a 5-item survey that assesses motivational readiness for adopting a consistent PA routine. Participants provide “Yes” or “No” responses to statements like, “I intend to become more physically active in the next 6 months.” [46]. Based on their responses, participants are classified as being in precontemplation, contemplation, preparation, action, or maintenance. Processes of Change for Physical Activity contains 40 items across 10 subscales assessing processes related to MVPA change that are either cognitive (eg, learning about the benefits of PA) or behavioral (eg, rewarding oneself for meeting goals) [46].

### Protocols

#### Overview

Once participants completed all baseline questionnaires and sufficient wear time was confirmed for the ActiGraph, the Fitbit monitors and apps were unblinded, and they were randomized 1:1 to a study condition. Randomization was done using REDCap clinical trials software, with participants stratified based on baseline stage of change to ensure equal distribution of motivational readiness for PA adoption across conditions. The randomization protocol was based on a permuted block-randomization protocol, with small, random-sized blocks. Friends or relatives were yoked into conditions together, with yoked groups distributed equally across conditions.

#### Tailored Mobile Health PA Intervention (Intervention Group)

A detailed description of the intervention has been published previously [28]. Summaries of the intervention components are included below.

##### Coaching session

The intervention consisted of a baseline counseling session (optional in-person or virtual) to teach key behavior change strategies, followed by theory-based components delivered via a range of remote technology channels over the next 6 months (details have been published previously) [28]. During the baseline visit, participants received a one-on-one health coaching session with a trained interventionist where they learned to set personalized activity goals. The coaching was grounded in motivational interviewing, and participants anticipated barriers and engaged in problem-solving techniques.

##### Study website

Participants in the intervention were also given an account on the study website, which included a range of personalized features. Participants entered weekly goals and scheduled their weekly activities. Their Fitbit data were synced with the website, which produced a calendar of their past activity and compared their activity with their set goals. Participants also filled out monthly questionnaires regarding their activity and psychosocial factors, which produced an individually tailored report on their activity progress and strategies for behavior change. Nonpersonalized features of the website included links to workout videos, solutions to common barriers, a list of places to be active including low-cost gyms, an interactive message board, and an activity leader board with the top 3 participants for the week based on current Fitbit data.

##### Text messages

Individually tailored automated algorithm-driven text messages based on participants’ Fitbit data were sent 4 times per week that updated them on the progress toward their goals and provided summaries of their MVPA for the week. Texts also prompted interactive goal setting and provided participants with goal progress feedback. Those who met their goals were congratulated and encouraged to continue increasing their goal; those who were not on track to meet their goal were given a Behavior Change Technique, such as goal setting, to try. Tailored texts also incorporated the names of friends and family and local places to be active.

##### Social Media

Participants were also invited to follow the intervention’s private Instagram account. Daily posts incorporated different theoretical constructs of behavior change from social cognitive theory and Behavior Change Techniques. Instagram stories were also used to prompt wearing and charging Fitbits.

Social media use was not required. Instagram posts were also shown on the study website for individuals who did not use social media.

At 1 week and 1 month postbaseline, a trained interventionist conducted a 10-minute phone call with participants to address any questions or concerns regarding the media channels or barriers to activity.

### Fitbit Only Group (Control Group)

The control group was told to continue wearing their Fitbit daily, and charge and sync regularly. Reminders to sync were sent via text message if a participant had not synced in 5 days. Participants in the control group received a US $10 Amazon gift card each month they remembered to wear, sync, and charge their Fitbit.

### Analysis

As a preliminary step, the study sample was summarized with respect to baseline sociodemographics and PA rates and compared between groups using *t* tests, chi-square tests, and nonparametrics as appropriate. Both participant and parent characteristics at baseline are reported for sociodemographics.

As minutes/week of MVPA were skewed and attempts to transform toward normality were unsuccessful, a series of quantile regression models with bootstrapped SEs (10,000 bootstrapped samples) were used to regress median minutes/week of MVPA on baseline values, group, and covariates. Models were run separately for self-reported MVPA (via the PAR) and objectively measured (using accelerometry). Models for the latter further adjusted for accelerometer wear time. As a small proportion of study participants were yoked together during the randomization process (siblings, cousins, or close friends who enrolled together), we adjusted for clustering by yoked group in each of our models (to ensure SEs were adjusting for nonindependence of outcomes within cluster). All other covariates were balanced at baseline and tested for significance in the model before being dropped.

To understand the group differences in minutes of PA as collected via the Fitbit, we examined data from the weeks corresponding to measurement of PA via the PAR and accelerometer. Using a longitudinal mixed effects model with subject-specific intercepts, we examined group differences adjusting for clustering by yoked pair.

All analyses were carried out in R (R Core Team), with significance level set at .05 a priori.

### Ethical Considerations

This study is approved by the University of California, San Diego Human Research Protections Programs (protocol #182070). Written, informed consent and parental permission and adolescent assent to participate are obtained from all participants. All data were deidentified for analyses. Fitbit devices and incentives in the form of cash payments and gift cards are provided to all participants, equating to US $25, US $50, and US $50 for baseline, 6-month, and 12-month follow-up measurement visits with an additional US $25 for each follow-up visit where all measures were completed.

## Results

**Figure 1.**
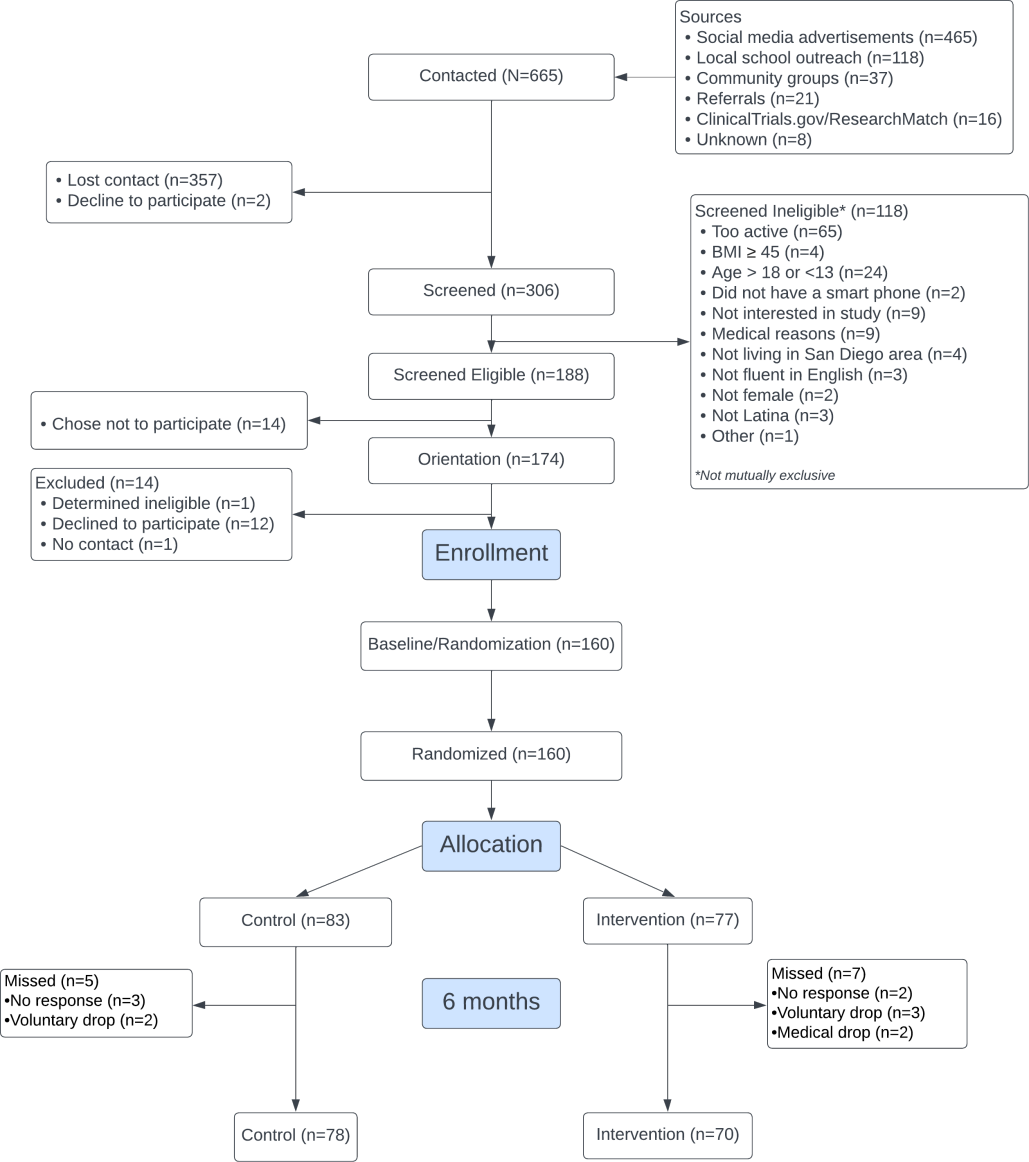
Six-month CONSORT (Consolidated Standards of Reporting Trials) diagram.

The study sample has been described in a previous publication [22]. In brief, participants were 15.85 years of age on average (SD 1.71), with an average BMI of 25.78 (SD 6.07). About half (84/160, 53%) reported speaking more English than Spanish, with 69% (110/160) of the aggregate sample reporting second-generation status. When considering motivation to become physically active, 84% (134/160) were considered in preparation. Participant retention at 6 months was high (148/160, 93%), with reasons for loss to follow-up depicted in Figure 1. More information on recruitment and challenges due to the COVID-19 pandemic is published elsewhere [22].

The parents’ sociodemographic data indicated that about half (86/160, 54%) of parents had a high school diploma, General Education Development (GED) equivalent, or less than high school level education, and about 25% of the sample reported an average household income of at least US $75,000. There were no between-group differences in baseline sociodemographics or psychosocial variables (all *P*>.05; see Table 1).

**Table 1.** Baseline characteristics of study sample by group (N=160).

Variables	Intervention (n=77)	Control (n=83)
Participant characteristics		
Age (years), mean (SD)	15.85 (1.71)	15.90 (1.50)
BMI, mean (SD)	26.16 (5.49)	25.24 (6.57)
Race, n (%)		
White	35 (45.5)	33 (39.8)
Black	1 (1.3)	0 (0.0)
Asian	1 (1.3)	1 (1.2)
American Indian/Alaska Native	1 (1.3)	2 (2.4%
Pacific Islander or Hawaiian	0 (0.0)	0 (0.0)
Multiracial	8 (10.4)	4 (48)
Other	31 (40.3)	43 (51.8)
Primary language, n (%)		
Spanish preferred over English	4 (5.2)	6 (7.2)
Spanish and English equally	21 (27.3)	31 (37.3)
English preferred over Spanish	47 (61.0)	37 (44.6)
Only English	5 (6.5)	9 (10.8)
Generational status, n (%)		
First generation	6 (7.8)	7 (8.4)
Second generation	53 (68.8)	57 (68.7)
Third generation	18 (23.4)	19 (22.9)
Parent characteristics		
Education, n (%)		
<High school	26 (33.8)	25 (30.1)
High school or GED	16 (20.8)	19 (22.9)
Some college or associate degree	14 (18.2)	18 (21.7)
College graduate	13 (16.9)	10 (12.0)
Master’s degree	8 (10.4)	8 (9.6)
Doctoral degree	0 (0)	3 (3.6)
Income (US dollar), n (%)		
<$11,999	12 (15.6)	22 (26.5)
$12,000-$24,999	12 (15.6)	11 (14.3)
$25,000-$49,999	21 (27.3)	21 (27.3)
$50,000-$99,999	21 (27.3)	18 (21.6)
$100,000+	11 (14.3)	11 (14.3)
Number of children in home, mean (SD)	2.01 (1.18)	1.93 (1.20)
Psychosocial constructs		
Neighborhood Environment (NEWS-Y)		
Land-use diversity, mean (SD)	2.47 (0.62)	2.37 (0.81)
Recreation, mean (SD)	2.27 (0.68)	2.21 (0.76)
Residential density, n (%)	112 (39.1)	109 (40.3)
Land-use access, mean (SD)	2.94 (0.50)	3.01 (0.50)
Street connectivity, mean (SD)	2.83 (0.59)	2.84 (0.57)
Walk or cycle facilities, mean (SD)	3.07 (0.59)	3.05 (0.66)
Neighborhood aesthetics, mean (SD)	2.74 (0.66)	2.89 (0.73)
Safety from traffic, mean (SD)	2.78 (0.45)	2.79 (0.45)
Safety from crime, mean (SD)	2.26 (0.78)	2.19 (0.75)
Neighborhood Environment (PANES), mean (SD)	4.96 (1.16)	4.59 (1.38)
PA Enjoyment (PACES), mean (SD)	4.67 (0.92)	5.03 (0.93)
Depressive symptoms (CES-D), mean (SD)	10.62 (3.87)	11.14 (4.70)
Exercise self-efficacy, mean (SD)	1.98 (0.54)	2.03 (0.46)
Friend social support for exercise, mean (SD)	20.5 (13.4)	24.2 (16.7)
Maternal support for exercise	2.46 (0.75)	2.55 (0.64)
Stage of change, n (%)		
Precontemplation	3 (3.9)	1 (1.2)
Contemplation	10 (13.0)	12 (14.5)
Preparation	64 (83.1)	70 (84.3)
Physical activity		
MVPA^b^ (min/wk), median (IQR)		
7-Day PAR^a^,^c^	119 (62.5-185)	120 (48.8-235)
ActiGraph GT3X+^a^	0 (0-24)	0 (0-26)
Total steps^a^, median (IQR)	5196 (3318-6634)	5175 (3536.12-6727.12)

a Variable had a skewed distribution. There were no significant between-group differences.

b MVPA: moderate-vigorous physical activity.

c PAR: Physical Activity Recall.

**Figure 2.**
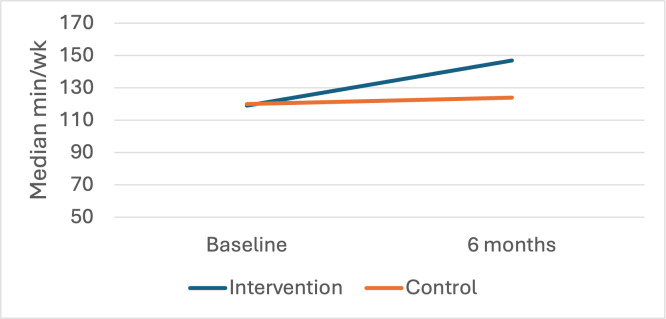
Between-group changes over time in moderate-vigorous physical activity (Physical Activity Recall).

Figures2  3 depict changes over time by group in unadjusted median minutes/week of MVPA from the PAR and accelerometer, respectively. The median minutes/week of self-reported MVPA among intervention participants increased from 119 (IQR 62.5-185) minutes/week at baseline to 147 (IQR 96-181) minutes/week at 6 months. Control participants showed increases from a median of 120 (IQR 48.8-235) at baseline to 124 (IQR 100-169) minutes/week at 6 months. Adjusted model results indicate intervention participants did an additional 23.5 minutes/week of MVPA at 6 months compared to control (95% CI 8.35‐32.76). Via accelerometry, intervention participants showed increases in minutes/week of MVPA from a median of 0 (IQR 0-24) minutes/week at baseline to 64 (IQR 19-72) minutes/week at 6 months compared to control participants, who showed increases from a median of 0 (IQR 0-26) at baseline to 41 (IQR 7-76) minutes/week at 6 months. Adjusted model results indicate intervention participants were doing an additional 18.4 minutes/week of MVPA at 6 months compared to control (95% CI 4.67‐37.28).

Finally, we considered daily steps (as measured by the Fitbit), restricted to weeks in which PAR and accelerometer data were also being collected (for comparability). At baseline, among the aggregated sample, the median daily steps were 5196 (IQR 3318-6634) among intervention participants and 5175 (IQR 3536.12-6727.12) among controls. Steps/day significantly increased in both arms with greater increases among intervention participants relative to control (β=101.25, SE=46.31; *P*=.03) suggesting intervention participants recorded an additional 101 steps/day compared to control at 6 months.

**Figure 3.**
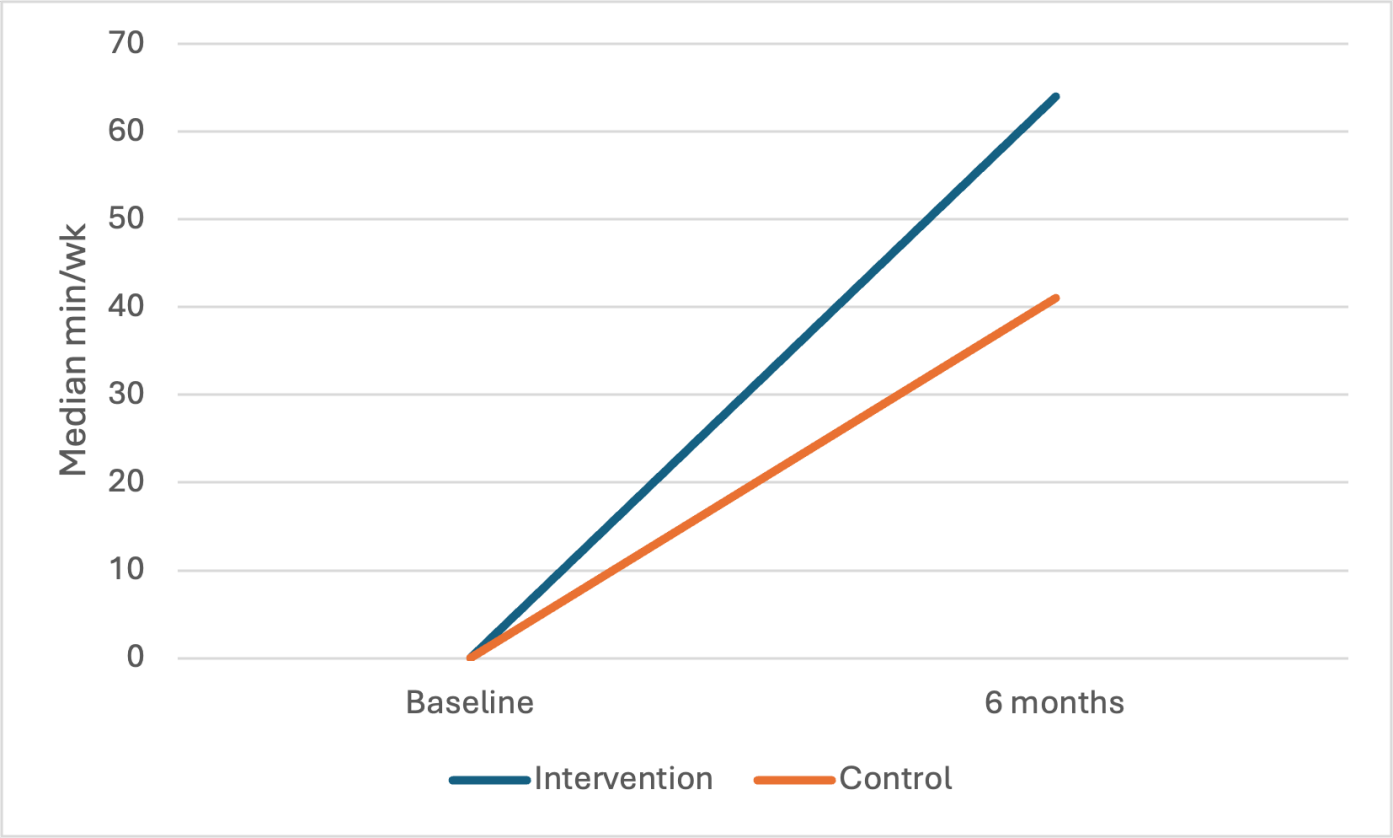
Between-group changes over time in moderate-vigorous physical activity (accelerometer).

## Discussion

### Principal Results

The aim of the Chicas Fuertes trial was to test the efficacy of a theory-based, individually tailored, multitechnology intervention for increasing MVPA in Latina adolescents. The results at 6 months presented here show statistically significant differences in changes to PA from baseline favoring the intervention group across objectively measured MVPA, self-reported MVPA, and daily steps. While both intervention and control conditions showed an increase in objectively measured PA from baseline, the intervention condition participated in an additional 18.4 minutes/week compared to the control condition. This effect was consistent with the self-reported data, with the intervention group reporting an additional 23.5 minutes/week compared to the controls, reinforcing the robustness of our findings.

### Comparison With Prior Work

Consistent with the existing body of literature [47], self-reported MVPA was consistently higher than objectively measured methods. While self-reported MVPA was higher at all time points for both the intervention and control groups, the magnitude of change measured by the ActiGraph was considerably larger, mostly due to the discrepancy between self-reported and objectively measured MVPA at baseline. This could suggest that participants did not have a clear understanding of what MVPA intensity felt like at baseline. This interpretation is consistent with previous research from this group that found significantly lower self-reported MVPA (which better correlated with Actigraph measured MVPA) when participants first engaged in a 10-minute demonstration walk at a moderate pace prior to conducting the PAR at baseline measurements [48]. Participants in this trial may have developed a better sense of MVPA over the course of the intervention; however, the simultaneous rise in MVPA minutes among the control condition suggests that access to the Fitbit and Fitbit App alone could be providing participants with better knowledge of MVPA.

The primary findings are consistent with previous research using similar intervention channels. Text-messaging interventions have been consistently effective at increasing PA in adults [49]. Specifically, meta-analyses suggested that interventions that used text-messaging in combination with other intervention components, such as in-person counseling sessions and websites, were more effective than those using text-messaging alone [49]. These findings are consistent with other research focused on adolescents with similar recommendations regarding the inclusion of goal setting and the combination with other web interfaces [50]. This paper is consistent with those findings by testing an approach that was both tailored to the participants and deployed multiple intervention components, such as coaching sessions, social media, and the website alongside text-messaging.

Despite health behavior and outcome disparities adversely impacting Latina adolescents, limited health behavior interventions have been explicitly designed for this population. This study begins to address that gap and builds on our previous results. Previous findings from our randomized controlled trials with adult Latina populations suggested that tailored technology-based interventions can be feasible and effective for increasing MVPA in adults [20,51-54]. The results here expand on this literature by further suggesting the efficacy of tailored technology-based interventions in Latina adolescents.

These findings are particularly significant given how sedentary the sample was at baseline, with a median of 0 (IQR 0-24) minutes/week of ActiGraph-measured MVPA. Increasing activity for individuals who were entirely sedentary, even if they do not meet PA guidelines, can have substantial impacts on their health risks [55]. Powell et al [55] posited that the benefits of activity are immediate and need no threshold to reduce the risk of all-cause mortality relative to those who are entirely inactive. Additionally, the greatest benefits to all-cause mortality risk are to those who go from no activity to any activity, further highlighting the importance of identifying interventions that are feasible and efficacious for inactive groups. As the most inactive demographic group, interventions that specifically improve health behaviors for Latina adolescents are needed to reduce health inequities and improve public health more generally [1]. This intervention provides evidence of effective, scalable, and contextually relevant strategies to increase MVPA among Latina adolescents.

### Limitations

Generalizability of these findings may be limited. The design was specifically chosen to target Latina adolescents in largely urban San Diego County; this precludes assumptions of this specific intervention strategy among other demographics, particularly among more rural communities where there may be fewer PA resources, thereby reducing intervention efficacy. Nearly 84% of the participants recruited were preparing to engage in more PA, so the effect of this intervention may not be as robust in groups who are less motivated to be active in the first place. Finally, these analyses only focused on MVPA as opposed to including LPA. While this is a common intervention target (and aligns with the 7-Day PAR), these findings preclude the ability to identify how LPA may be important for participants who are trending from inactive to more active lifestyles.

Despite limitations, this study has numerous strengths, notably the randomized controlled trial design. It directly addresses a gap in the literature by testing a technology-based intervention to improve PA in Latina adolescents. The success of this intervention can be partially attributed to multiple steps of iterative codesign with the target population [22,28]. This study also had a high retention rate (93%), further signaling the acceptability and feasibility of our approach. Additionally, the control condition was an active comparator that received a Fitbit monitor, allowing us to compare our intervention to a commercially available program. Our approach used multiple validated measures of PA providing robustness for our primary accelerometer-based findings. Finally, the study was conducted during the COVID-19 pandemic, showing that the intervention can be effective even amid marked interruptions to daily routines.

### Conclusions

This intervention was successful in using a tailored technology-based strategy to increase MVPA in Latina adolescents and provides a promising and scalable method for addressing a key health behavior. This study was co-designed with the target population, intentionally using digital media channels with the potential for broad scale-up and remote delivery. Future studies should examine strategies for dissemination and implementation, including partnering with educational and clinical organizations.

## Supplementary material

10.2196/71623 Checklist 1 CONSORT-eHEALTH checklist (V 1.6.1).

## Abbreviations

CES-D: Center for Epidemiological Studies Depression Scale
CONSORT: Consolidated Standards for Reporting Trials
GED: General Education Development
HIPAA: Health Insurance Portability and Accountability Act
LPA: light physical activity
MVPA: moderate-vigorous physical activity
NEWS-Y: Neighborhood Environment Walkability Scale for Youth
PA: physical activity
PACES: Physical Activity Enjoyment Scale
PANES: Physical Activity Neighborhood Environment Scale
PAR: Physical Activity Recall
REDCap: Research Electronic Data Capture
